# Co-infection in pediatric pertussis during 2023 and 2024 in southern China: pathogen distribution characteristic and influence on manifestation of pertussis

**DOI:** 10.3389/fmicb.2025.1722003

**Published:** 2025-12-03

**Authors:** Weiming Lai, Junfei Guo, Xiaoying Zhou, Yasha Luo, Minling Zheng, Qiongdan Mai, Jiana Xiong, Mingyong Luo

**Affiliations:** 1 Department of Clinical Laboratory, Guangdong Women and Children Hospital, Guangzhou, China; 2 Guangzhou Medical University, Guangzhou, China; 3 Women and Children’s Hospital, Southern University of Science and Technology, Shenzhen, China

**Keywords:** pertussis, children, co-infection, symptoms, China

## Abstract

**Objective:**

We sought to examine the pathogen composition of co-infections in pertussis during 2023 and 2024 and assess the impacts of co-infection on pertussis.

**Methods:**

Clinical data of pediatric pertussis patients were retrospectively analyzed. The impact of co-infections on pertussis, the detection rate and composition of co-pathogens were analyzed. Culture, polymerase chain reaction, or metagenomic sequencing were used in pertussis or co-pathogens detection.

**Results:**

Among 620 pertussis patients, 110 patients co-infected with bacteria, 106 co-infected with virus, and 98 co-infected with both bacteria and virus. *M. pneumoniae* (114, 18.39%), *M. catarrhalis* (44, 7.1%), and *H. influenzae* (26, 4.19%) were the most common bacterial co-pathogens, and *Rhinovirus* (100, 16.12%), *Cytomegalovirus* (31, 5%), and *Influenza virus A&B* (28, 4.52%) were the most common viral co-pathogens. Patients with co-infections exhibited more severe manifestations than those with pertussis alone, and the severity was associated with the type and number of pathogens. The composition of co-pathogens linked to the age and hospitalization status of the patients. *M. pneumoniae* (91, 21.06%), *M. catarrhalis* (32, 7.41%), and *Influenza virus A&B* (22, 5.09%) primarily caused co-infections in older, non-hospitalized patients, whereas *Cytomegalovirus* [(19, 14.39%) non-ICU, (12, 21.43%) ICU] and *Respiratory syncytial virus* [(8, 6.06%) non-ICU, (8, 14.28%) ICU] were more common in younger, hospitalized patients.

**Conclusion:**

Co-infections are common among pertussis and can intensify clinical symptoms. Distribution of co-infecting pathogens is associated with age and hospitalization status, and the impacts of co-infection on the manifestations of pertussis varied according to the types and number of co-pathogens.

## Introduction

Pertussis is a highly contagious respiratory infectious disease that can be prevented by vaccination (Kilgore et al., 2016; Domenech de Cellès and Rohani, 2024). The widespread use of pertussis vaccines globally has significantly reduced disease incidence and mortality rates (Domenech de Cellès and Rohani, 2024). However, in recent years, there has been a “resurgence of pertussis” in many regions worldwide, posing a serious health threat to children, particularly infants who have not been vaccinated against pertussis (Monchausse et al., 2025; Tian et al., 2025). The characteristic features of pertussis include paroxysmal spasmodic coughing, inspiratory whooping at the end of coughing, and post-tussive vomiting (Nieves and Heininger, 2016). However, clinical manifestations vary depending on factors such as age, immune status, infection history, and disease progression, and atypical clinical symptoms may lead to delayed diagnosis and treatment, affecting prognosis. Pertussis is more likely to co-infect other pathogens than other bacterial infections (Korppi and Hiltunen, 2007; Cosnes-Lambe et al., 2008; Pandolfi et al., 2021). However, whether co-infection intensifies or alters the clinical symptoms of pertussis remains inconclusive (Frassanito et al., 2017; Muloiwa et al., 2020; Gan and Wu, 2025; Qing et al., 2025; Scutari et al., 2025). According to comprehensive literature reports, the composition of co-infecting pathogens in pertussis varies significantly across different regions and years, and the impacts of co-infection on pertussis symptoms and prognosis are considerably different (Gan and Wu, 2025; Qing et al., 2025; Scutari et al., 2025; Tian et al., 2025). The COVID-19 pandemic has led to significant changes in the respiratory pathogen spectrum in many regions worldwide, including China (Li Q. et al., 2024; Du et al., 2025). However, there is limited research on whether these changes affect the spectrum of co-infecting pathogens in pertussis, which further affecting the manifestation of pertussis.

Our study will examine the co-infection status of children with pertussis in our hospital from 2023 to 2024, explore how co-infection affects clinical symptoms and laboratory test results, and establish the effects of various types of co-infecting pathogens and different number of co-infecting pathogens on pertussis symptoms. We will also further clarify the composition of co-infecting pathogens in patients with pertussis across different age groups and hospitalization statuses.

## Materials and methods

### Study design

This retrospective cross-sectional study aimed to reveal the prevalence of co-infection and related pathogens among pediatric patients with pertussis during 2023 and 2024. The clinical manifestations and laboratory findings of patients with different co-infection situations were then comprehensively analyzed. The study was conducted at Guangdong Women and Children Hospital, a tertiary women and children hospital in Guangzhou, Guangdong Province, China. The study subjects were patients with pertussis, aged ≤ 14-year-old, with complete medical records. The Medical Ethics Committee of our institute approved our study (approval number20251008) and the study complied with the *Declaration of Helsinki*. Informed consent was waived due to the retrospective design of the study, which was approved by our institute’s Medical Ethics Committee.

### Data collection

The basic information, clinical symptoms, and laboratory test results of the enrolled subjects were collected through our medical record and laboratory information system. The basic information collected included the patient’s gender and age. The clinical data collected included the patient’s diagnosis, pertussis-related symptoms (e.g., paroxysmal coughing, post-tussive vomiting, cyanosis, etc.), fever status, and hospitalization status. Laboratory test results primarily included peripheral white blood cell count, neutrophil and lymphocyte counts, blood hs-CRP and PCT levels at the first medical visit, and pathogen detection results. Neutrophil-to-lymphocyte ratios (NLR) were calculated according to neutrophil and lymphocyte counts. Pertussis was detected using culture (OXID, United Kingdom), polymerase chain reaction (Da An Gene Co., China) or metagenomic sequencing (Hua Da Gene Co., China), using nasopharyngeal swab samples or bronchoalveolar lavage fluid. Co-infection pathogens were detected using methods such as metagenomic sequencing, multiplex PCR, culture, or antibody testing. The sample collection time for co-infection pathogen testing was the same or within 24 h of the sampling time of the pertussis-positive sample. All data were securely stored to prevent unauthorized access to patient privacy, with anonymized patient personal information during data processing.

### Grouping method

To clarify the impact of co-infections on the clinical symptoms and laboratory test results of pertussis, the study participants were divided into a pertussis mono-infection group and a pertussis co-infection group based on the presence or absence of co-infections. The groups were compared in terms of gender distribution, age, clinical symptoms, and laboratory test results. To further elucidate the effects of bacterial and viral co-infections on the clinical symptoms and laboratory test results of patients with pertussis, we categorized pertussis with co-infections into a bacterial co-infection group, a viral co-infection group, and a bacterial/viral co-infection group. To examine the effects of various numbers of co-infecting pathogens on the clinical symptoms of pertussis, the pertussis with co-infections were divided into a single-pathogen co-infection group, a two-pathogen co-infection group, and a three-or-more-pathogen co-infection group. The children were also divided into the following groups based on age: < 2, 2–6, 6–12, and > 12 months. The detection rates of different pathogens across these age groups were compared, and the composition of co-infecting pathogens in children of different age groups was analyzed. Children were categorized into an outpatient group, a non-ICU inpatient group, and an ICU inpatient group based on hospitalization status. Comparison was made between the detection rates of various pathogens across different hospitalization status groups, and the composition of co-infecting pathogens was analyzed in children with distinct hospitalization statuses.

### Statistical analysis

GraphPad Prism version 10.0 was used to generate the figures and conduct relevant statistical analyses. Continuous variables were expressed as mean ± SD, and compared using *t*-tests or one-way ANOVA if normally distributed, or median [Q1, Q3], and compared using Mann-Whitney tests or Kruskal-Wallis test if non-normally distributed. Categorical variables were expressed as frequency (%), and chi-squared tests or Fisher’s exact tests were used for comparison. *p* < 0.05 was considered statistically significant.

## Results

### Patient characteristics and comparison between patients with and without co-infection

As shown in Table 1, 620 pediatric pertussis cases met the inclusion criteria during the study period. Among them, 273 (44.03%) were female. Most cases occurred in children over 12 months of age (416, 67.09%), followed by children aged 2–6 months (131, 21.13%). Of the 620 patients with pertussis, 188 (30.33%) required hospitalizations, and 56 (9.03%) were admitted to the ICU. The median length of hospital stay was 8 days (IQR: 6, 12), and the median length of ICU stay was 13 days (IQR: 9, 19). Paroxysmal cough was the most common clinical symptom, observed in 408 (65.81%) children, with a median cough duration of 7 days (IQR: 4, 14). Fever was present in 210 (33.87%) patients, facial flushing in 173 (27.90%), and post-tussive vomiting in 133 (21.45%). Cyanosis and whooping were less frequent, occurring in only 71 (11.45%) and 47 (7.58%) patients, respectively. Forty-three patients, representing 6.94%, needed assistance ventilation, with 17 patients, accounting for 2.74%, receiving invasive mechanical ventilation. The median peripheral white blood cell (WBC) count was 11.06 × 10^9^/L (IQR: 8.21 × 10^9^/L, 15.72 × 10^9^/L), the median neutrophil count was 4.26 × 10^9^/L (IQR: 2.95 × 10^9^/L, 6.56 × 10^9^/L), and the median lymphocyte count was 4.77 × 10^9^/L (IQR: 2.95 × 10^9^/L, 8.39 × 10^9^/L). The median hsCRP level was 0.45 mg/L (IQR: 0.2, 3.1), and the median PCT level was 0.25 ng/mL (IQR: 0.31, 0.4).

**TABLE 1:** Characteristics of patients and comparisons between patients with and without co-infection.

Items	Overall (*N* = 620)	Pertussis only (*N* = 306)	Pertussis with co-infection (*N* = 314)	*p*
**Gender**				
Female, N (%)	273 (44.03%)	147 (48.04%)	126 (40.12%%)	0.0522
**Age, N (%)**				
< 2 M	43 (6.94%)	20 (46.51%)	23 (53.49%)	0.0102
2–6 M	131 (21.13%)	49 (37.4%)	82 (62.6%)	
6–12 M	30 (4.84%)	24 (80%)	6 (20%)	
> 12 M	416 (67.09%)	220 (52.88%)	196 (47.12%)	
**Hospitalization status, N (%)**				
Outpatient	432 (69.67%)	251 (82.03%)	181 (57.64%)	< 0.0001
Inpatient	188 (30.33%)	55 (17.97%)	133 (42.36%)	
Length of stay, median (IQR)	8 (6, 12)	7 (4, 10)	10 (6,14)	0.0003
ICU admission, N (%)	56 (9.03%)	13 (4.25%)	43 (13.69%)	< 0.0001
Length of ICU stay, median (IQR)	13 (9–19)	11 (6.5, 16)	14 (10, 20)	0.0997
**Clinical manifestations, N (%)**				
Paroxysmal cough	408 (65.81%)	187 (61.11%)	221 (70.38%)	0.0177
Length of cough, median (IQR)	7 (4, 14)	7 (4, 14)	8 (4, 15)	0.0321
Inspiratory whooping	47 (7.58%)	21 (6.86%)	26 (8.28%)	0.546
Facial flushing	173 (27.90%)	80 (26.14%)	93 (29.62%)	0.3707
Cyanosis	71 (11.45%)	23 (7.52%)	48 (15.29%)	0.0024
Posttussive emesis	133 (21.45%)	64 (20.91%)	69 (21.97%)	0.7698
Fever	210 (33.87%)	84 (27.45%)	126 (40.13%)	0.0009
**Laboratory findings**				
Leukocyte, median (IQR), *10^9^/L	11.06 (8.21, 15.72)	10.67 (8.14, 14.16)	11.69 (8.3, 17.78)	0.0183
Neutrophils, median (IQR), *109/L	4.26 (2.95, 6.56)	3.96 (2.74, 5.53)	4.66 (3.08, 7.5)	0.0003
Lymphocyte, median (IQR), *109/L	4.77 (2.95, 8.39)	4.94 (3.06, 7.99)	4.67 (2.72, 9.51)	0.8814
NLR, median (IQR)	0.85 (0.41, 1.74)	0.79 (0.4, 1.46)	1.02 (0.42, 2.05)	0.0229
hsCRP mg/L median (IQR)	0.45 (0.2, 3.1)	0.28 (0.2, 1.59)	0.76 (0.21, 4.71)	< 0.0001
PCT ng/ml median (IQR)	0.25 (0.31, 0.4)	0.31 (0.25,0.38)	0.32 (0.24, 0.41)	0.9347
**Ventilation status, N(%)**				
With assisted ventilation	43 (6.94%)	9 (2.94%)	34 (10.83%)	0.0001
Non-invasive ventilation	26 (4.19%)	6 (1.96%)	21 (6.69%)	0.005
Invasive ventilation	17 (2.74%)	3 (0.98%)	13 (4.14%)	0.0198

As presented in Table 1, there was no significant difference in the gender distribution between the pertussis-only infection group and co-infection group. Patients with pertussis under 6 months of age were more likely to have co-infections. Those with co-infections had significantly higher rates of hospitalization (42.36% vs. 17.97%), longer hospital stays, higher ICU admission rates (13.69% vs. 4.25%), and longer ICU stays than those with pertussis-only infection. A greater proportion of children with co-infections exhibited symptoms such as paroxysmal cough (70.38% vs. 61.11%), cyanosis (15.29% vs. 7.52%), and fever (40.13% vs. 27.45%) relative to the pertussis-only group. More patients in the co-infection group required assisted ventilation (10.83% vs. 2.94%). In addition, the peripheral WBC counts, neutrophil counts, and NLR were significantly higher in the co-infection group, as were the hsCRP levels, compared to the pertussis-only group.

### Comparisons between patients co-infected with different kinds of pathogens

According to Table 2, of the 314 pertussis patients with co-infections, 110 (35.03%) had bacterial co-infection alone, 106 (33.75%) had viral co-infection alone, and 98 (31.22%) had both bacterial and viral co-infections. No notable variations were found in gender distribution across the various co-infection groups. Regarding age composition, all groups were predominantly composed of children aged 2–6 months and over 12 months. The proportion of patients requiring hospitalization and ICU admission was highest in the group with both viral and bacterial co-infections, followed by the viral co-infection group. Except for paroxysmal cough and cyanosis, no significant differences in clinical symptoms were observed between the groups. The incidence of paroxysmal cough and cyanosis was highest in the group with both viral and bacterial co-infections, followed by the group with only viral co-infection. The proportion of patients needing assisted ventilation was substantially higher in the group with dual infections of both viruses and bacteria compared to the other two groups. Patients with viral co-infections had significantly higher peripheral white blood cell and lymphocyte counts compared to those with bacterial co-infections alone, but no notable differences were observed in other laboratory test outcomes between the groups.

**TABLE 2:** Comparisons between patients co-infected with different kinds of pathogens.

Items	Co-infection with bacteria (*N* = 110)	Co-infection with virus (*N* = 106)	Co-infection with bacteria and virus (*N* = 98)	*P*
**Gender**				
Female, N (%)	70 (63.64%)	54 (50.94%)	62 (63.27%)	0.1093
**Age, N (%)**				
< 2 M	6 (5.45%)	10 (9.43%)	8 (8.16%)	0.9768
2–6 M	14 (12.73%)	39 (36.79%)	28 (28.57%)	
6–12 M	3 (3.64%)	3 (2.83%)	7 (7.14%)	
> 12 M	87 (79.09%)	54 (50.94%)	55 (56.12%)	
**Hospitalization status, N (%)**				
Outpatient	68 (70.915)	56 (52.83%)	42 (42.86%)	0.0016
Inpatient	32 (29.09%)	50 (47.17%)	56 (57.14%)	
Length of stay, median (IQR)	9 (5, 13)	10 (7, 13)	10 (7, 15)	0.7141
ICU admission,N(%)	8 (7.27%)	17 (16.04%)	22 (22.45%)	0.0057
Length of ICU stay, median (IQR)	14 (7, 23)	12 (9,19)	14 (10,20)	0.6271
**Clinical manifestations, N (%)**				
Paroxysmal cough	66 (60%)	78 (73.58%)	78 (79.59%)	0.0066
Length of cough, median (IQR)	10 (4, 15)	8 (5, 15)	7 (4, 15)	0.924
Inspiratory whooping	7 (6.36%)	11 (10.38%)	7 (7.14%)	0.5483
Facial flushing	26 (23.64%)	37 (34.91%)	28 (28.57%)	0.1905
Cyanosis	11 (10%)	18 (16.98%)	22 (22.45%)	0.0413
Posttussive emesis	19 (17.27%)	32 (30.19%)	22 (22.45%)	0.087
Fever	42 (38.18%)	38 (35.85%)	47 (47.96%)	0.2066
**Laboratory findings**				
Leukocyte, median (IQR), *10^9^/L	10.17 (7.63, 14.44)	12.58 (8.55, 20.19)	12.39 (8.58, 18.65)	0.0438
Neutrophils, median (IQR), *109/L	4.49 (3.04, 6.48)	4.79 (3.03, 7.35)	4.82 (3.3, 8.65)	0.3119
Lymphocyte, median (IQR), *109/L	4.16 (2.85, 5.77)	6.21 (3.2, 12.06)	5.05 (2.21, 8.89)	0.0455
NLR, median (IQR)	1.1 (0,55, 2.09)	0.7 (0.35, 1.65)	1.28 (0.46, 2.78)	0.0765
hsCRP mg/L	0.54 (0.2, 5.17)	0.51 (0.2, 3.65)	1.87 (0.38, 5.36)	0.0672
PCT ng/mL	0.31 (0.24, 0.36)	0.33 (0.24, 0.44)	0.32 (0.25, 0.42)	0.2256
**Ventilation status, N(%)**				
With assisted ventilation	8 (7.27%)	10 (9.43%)	19 (19.39%)	0.0147
Non-invasive ventilation	3 (2.73%)	6 (5.66%)	11 (11.22%)	0.0461
Invasive ventilation	5 (4.55%)	4 (3.77%)	8 (8.16%)	0.3821

### Comparisons between patients co-infected with different numbers of pathogens

As shown in Table 3, among 314 pertussis patients with co-infections, 178 (56.68%) were co-infected with a single pathogen, 82 (26.11%) with two pathogens, and 54 (17.19%) with three or more pathogens. No significant differences were observed in gender or age distribution among patients with different numbers of co-infecting pathogens. The proportion of patients requiring hospitalization and ICU admission increased significantly as the number of co-infecting pathogens increased. The incidence of paroxysmal cough and cyanosis also increased with a greater number of co-infecting pathogens, while no significant association was found between the number of co-pathogens and other clinical symptoms. The need for assisted ventilation increased with the number of co-infecting pathogens. However, the number of co-infecting pathogens had no significant impact on the results of laboratory tests.

**TABLE 3:** Comparisons between patients co-infected with different number of pathogens.

Items	One co-infection (*N* = 178)	Two co-infections (*N* = 82)	Three or more co-infections (*N* = 54)	*P*
**Gender**				
Female, N (%)	97 (54.49%)	50 (60.98%)	38 (70.37%)	0.1052
**Age, N (%)**				
< 2 M	12 (6.74%)	8 (9.76%)	3 (5.56%)	0.1417
2–6 M	39 (21.91%)	24 (29.26%)	19 (35.19%)	
6–12 M	7 (3.93%)	1 (1.22%)	5 (9.26%)	
>12 M	120 (67.42%)	49 (59.76%)	27 (50%)	
**Hospitalization status, N (%)**				
>Outpatient	124 (69.66%)	39 (47.56%)	13 (24.07%)	< 0.0001
>Inpatient	54 (30.34%)	43 (52.44%)	41 (75.93%)	
>Length of stay, median (IQR)	9 (6, 14)	10 (7, 15)	10 (7, 14)	0.9224
>ICU admission,N(%)	12 (6.74%)	18 (21.95%)	26 (48.15%)	< 0.0001
>Length of ICU stay, median (IQR)	17 (9, 21)	13 (10,18)	14 (10, 20)	0.5316
**Clinical manifestations, N (%)**				
>Paroxysmal cough	114 (64.04%)	63 (76.83%)	44 (81.48%)	0.0167
>Length of cough, median (IQR)	7 (4, 15)	8 (4, 15)	10 (5, 20)	0.7446
Inspiratory whooping	15 (8.43%)	2 (4.88%)	5 (9.26%)	0.1468
Facial flushing	47 (26.4%)	28 (34.15%)	15 (27.78%)	0.4283
Cyanosis	17 (9.55%)	15 (18.29%)	18 (33.33%)	0.0002
Posttussive emesis	39 (21.91%)	22 (26,83%)	11 (20.37%)	0.6072
Fever	62 (34.83%)	39 (47.56%)	25 (46.3%)	0.0906
**Laboratory findings**				
Leukocyte, median (IQR), *10^9^/L	10.85 (7.83, 17.08)	12.58 (8.94, 17.81)	12.37 (8.02, 18.58)	0.6011
Neutrophils, median (IQR), *109/L	4.65 (3.06, 6.94)	5.17 (3.31, 9.58)	4.06 (2.88, 5.99)	0.0856
Lymphocyte, median (IQR), *109/L	4.23 (2.74, 9.69)	4.76 (2.67, 8.23)	6.05 (2.44, 10.04)	0.9841
NLR, median (IQR)	1.04 (0.43, 1.94)	1.08 (0.45, 2.46)	0.65 (0.32, 2.09)	0.163
hsCRP mg/L	0.51 (0.2, 4.37)	2.08 (0.39, 6.56)	0.63 (0.2, 3.93)	0.4126
PCT ng/mL	0.32 (0.24, 0.38)	0.32 (0.24, 0.44)	0.35 (0.25, 0.44)	0.0822
**Ventilation status, N(%)**				
With assisted ventilation	10 (5.62%)	13 (18.85%)	13 (24, 07%)	0.0003
Non-invasive ventilation	4 (2.25%)	10 (12.19%)	9 (16.67%)	0.0001
Invasive ventilation	6 (3.37%)	3 (3.66%)	4 (7.41%)	0.4162

### Distribution of co-infection pathogen according to patient age

As shown in Table 4, 452 co-infecting pathogens were detected in 314 children with mixed infections. *M. pneumoniae*, *M. catarrhalis*, and *H. influenzae* were the three most frequently detected bacterial co-pathogens, while *Rhinovirus*, *Cytomegalovirus*, and *Influenza virus A&B* were the three most common viral co-pathogens. *Candida* spp. and *P. jirovecii* were the only fungal pathogens identified.

**TABLE 4:** The detection rate of pathogens across different age groups.

Items	Overall (*N* = 620)	Age < 2 M (*N* = 43)	Age 2–6 M (*N* = 131)	Age 6–12 M (*N* = 30)	Age > 12 M (*N* = 416)
**Bacteria, N (detection rate)**					
*Mycoplasma pneumoniae*	114 (18.39%)	1 (2.33%)	9 (6.87%)	1 (3.33%)	103 (24.76%)
*Moraxella catarrhalis*	44 (7.1%)	1 (2.33%)	1 (0.76%)	0	42 (10.1%)
*Haemophilus influenzae*	26 (4.19%)	2 (4.65%)	13 (9.92%)	0	11 (2.64%)
*streptococcus pneumoniae*	13 (2.1%)	2 (4.65%)	3 (2.29%)	1 (3.33%)	7 (1.68%)
*Escherichia coli*	7 (1.13%)	3 (6.98%)	2 (1.53%)	0	2 (0.48%)
*Klebsiella pneumoniae*	7 (1.13%)	2 (4.65%)	2 (1.53%)	0	3 (0.72%)
*Staphylococcus aureus*	11 (1.77%)	2 (4.65%)	4 (3.05%)	5 (16.67%)	0
*Acinetobacter baumannii*	2 (0.32%)	0	1 (0.76%)	0	1 (0.24%)
*Pseudomonas aeruginosa*	2 (0.32%)	1 (2.33%)	1 (0.76%)	0	0
*Chlamydia pneumoniae*	1 (0.16%)	0	0	0	1 (0.24%)
**Virus, N (detection rate)**					
*Rhinovirus*	100 (16.12%)	3 (6.98%)	30 (22.9%)	5 (16.67%)	62 (14.9%)
*Cytomegalovirus*	31 (5%)	5 (11.63%)	18 (13.76%)	0	8 (1.92%)
*Influenza virus A&B*	28 (4.52%)	0	1 (0.76%)	0	27 (6.49%)
*Respiratory syncytial virus*	17 (2.74%)	6 (11.95%)	8 (6.11%)	0	3 (0.72%)
*Herpes viruses*	14 (2.26%)	0	2 (1.53%)	0	12 (2.88%)
*Adenovirus*	12 (1.94%)	0	3 (2.29%)	0	9 (2.16%)
*ParaInfluenza virus*	10 (1.61%)	1 (2.33%)	1 (0.76%)	1 (3.33%)	7 (1.68%)
*Human metapneumovirus*	7 (1.13%)	0	2 (1.53%)	0	5 (1.2%)
**Fungus, N (detection rate)**					
*Candida*	8 (1.29%)	0	2 (1.53%)	0	6 (1.44%)
*Pneumocystis jirovecii*	5 (0.81%)	0	4 (3.05%)	0	1 (0.24%)

**FIGURE 1:**
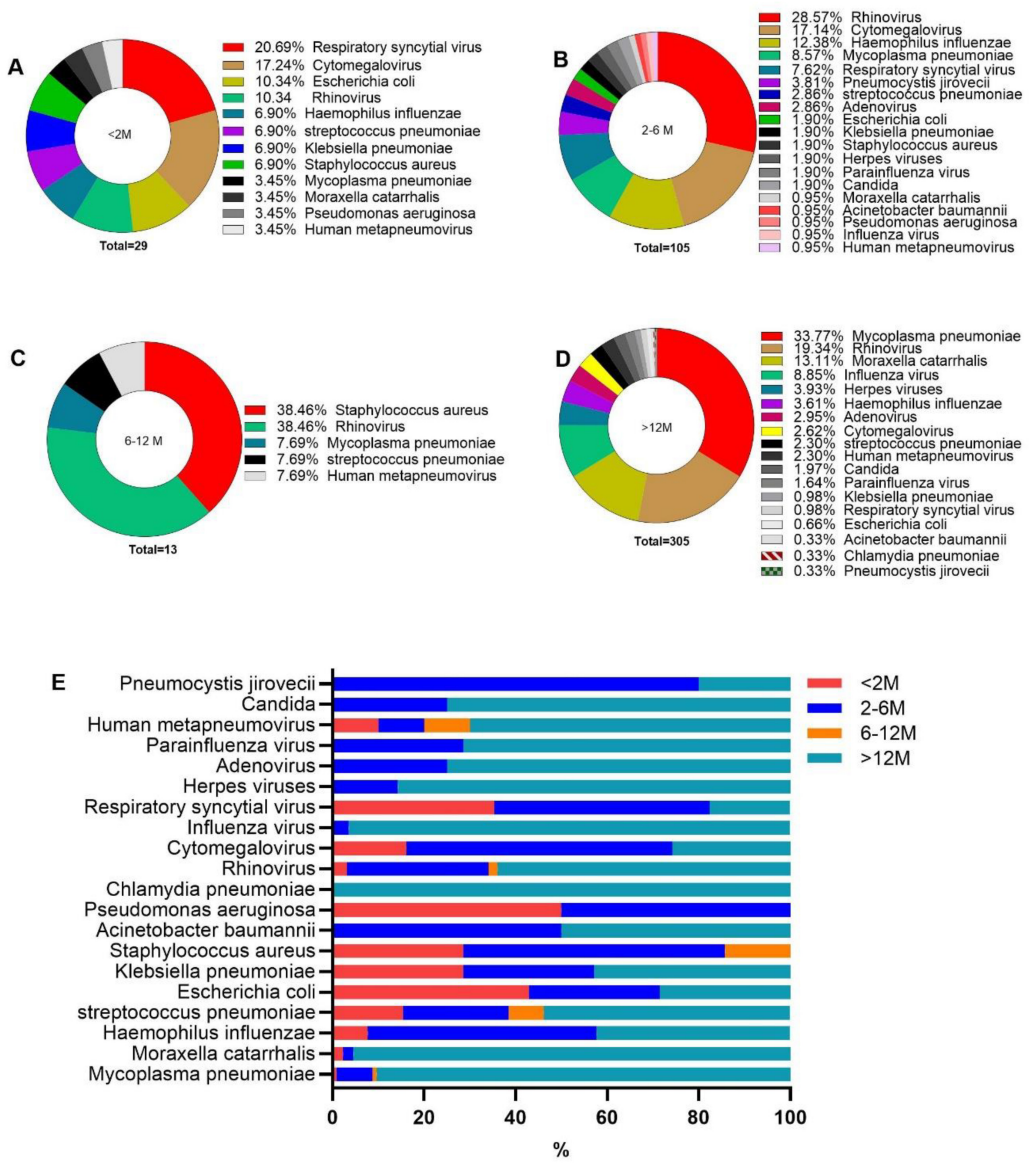
The composition of co-infected pathogens of different age groups. **(A–D)** The composition of co-infected pathogens in pertussis patients of different age groups as indicated. **(E)** Age distribution of different co-infected pathogens.

Significant differences in the detection rates and distribution of co-infecting pathogens were observed across different age groups (Table 4; Figure 1). *Cytomegalovirus* and *Respiratory syncytial virus* were detected significantly more frequently in infants under 6 months of age than in older children. In contrast, *M. pneumoniae*, *M. catarrhalis*, and *Influenza virus A&B* were significantly more prevalent in children over 12 months of age compared with other groups. *P. jirovecii* was primarily isolated from pertussis patients aged 2–6 months.

### Distribution of co-infection pathogen according to hospitalization status

**FIGURE 2:**
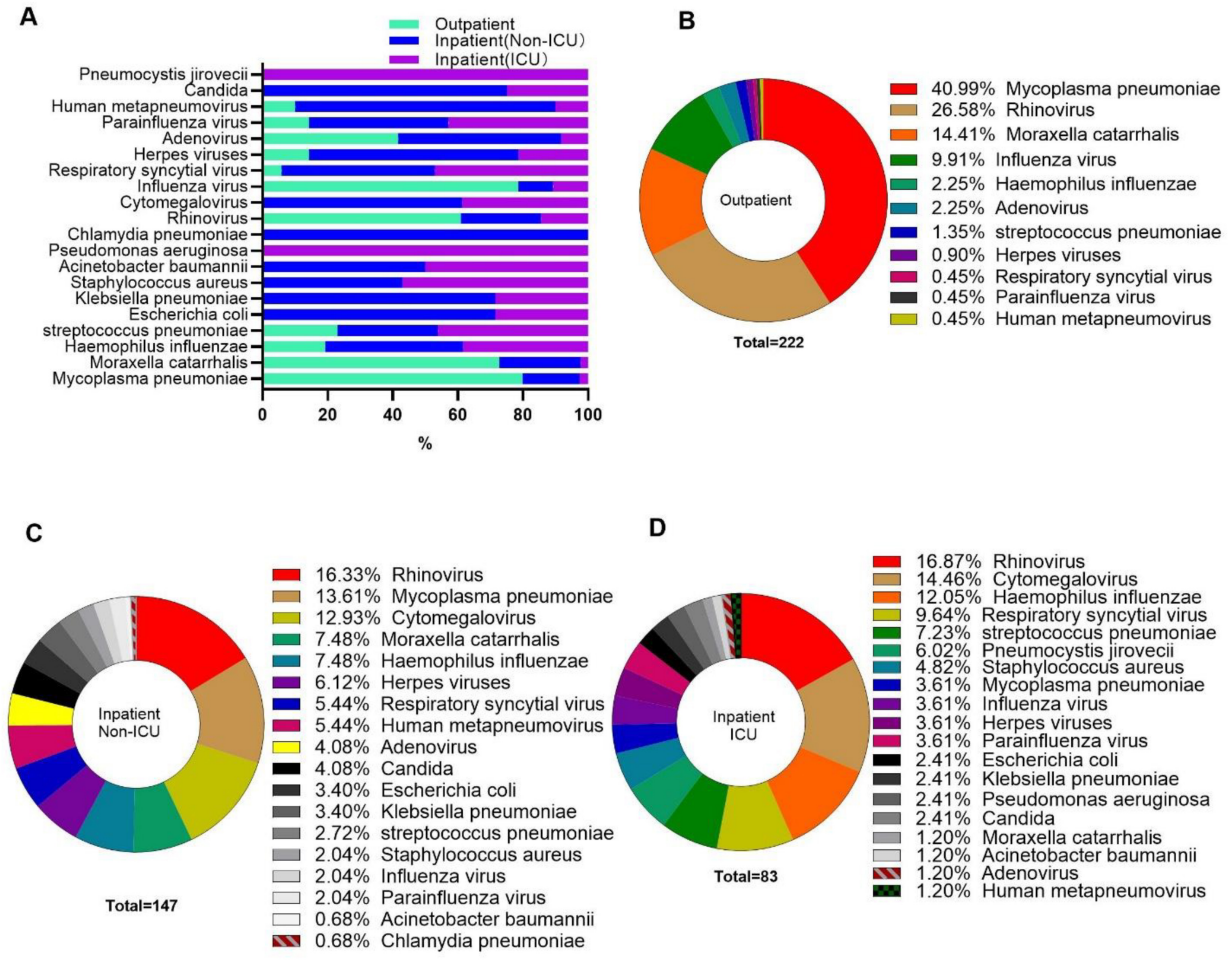
The composition of co-infected pathogens of patients with different hospitalization status. **(A)** The distribution of different co-infected pathogens according to hospitalization status. **(B–D)** The composition of co-infected pathogens in pertussis patients with different hospitalization status as indicated.

Observations were made regarding substantial discrepancies in the detection rates and the distribution of co-infecting pathogens among *patients with pertussis who had different hospitalization statuses* (Table 5 and Figure 2). *M. pneumoniae*, *M. catarrhalis*, and *H. influenzae* were the most common bacterial coinfections among outpatients and non-ICU inpatients. In contrast, ICU inpatients were primarily co-infected with *H. influenzae*, *S. pneumoniae*, and *M. pneumoniae*. *Rhinovirus* and *Influenza virus A&B* were the predominant viral co-pathogens in outpatients, whereas *Cytomegalovirus* and *Respiratory syncytial virus* were more frequently detected in hospitalized patients. Fungal co-infections were mainly identified in inpatients, and all cases of *P. jirovecii* were isolated from patients admitted to the ICU.

**TABLE 5:** The detection rate of pathogens among patients with different hospitalization status.

Items	Outpatient (*N* = 432)	Inpatient (Non-ICU, *N* = 132)	Inpatient (ICU, *N* = 56)
**Bacteria, N (detection rate)**			
*Mycoplasma pneumoniae*	91 (21.06%)	20 (15.15%)	3 (5.36%)
*Moraxella catarrhalis*	32 (7.41%)	11 (8.33%)	1 (1.79%)
*Haemophilus influenzae*	5 (1.16%)	11 (8.33%)	10 (17.86%)
*Streptococcus pneumoniae*	3 (0.69%)	4 (3.03%)	6 (10.71%)
*Escherichia coli*	0	5 (3.79%)	2 (3.57%)
*Klebsiella pneumoniae*	0	5 (3.79%)	2 (3.57%)
*Staphylococcus aureus*	0	3 (2.27%)	4 (7.14%)
*Acinetobacter baumannii*	0	1 (0.76%)	1 (1.79%)
*Pseudomonas aeruginosa*	0	0	2 (3.57%)
*Chlamydia pneumoniae*	0	1 (0.76%)	0
**Virus, N (detection rate)**			
*Rhinovirus*	59 (13.66%)	24 (18.18%)	14 (25%)
*Cytomegalovirus*	0	19 (14.395)	12 (21.43%)
*Influenza virus A&B*	22 (5.09%)	3 (2.27%)	3 (5.36%)
*Respiratory syncytial virus*	1 (0.23%)	8 (6.06%)	8 (14.28%)
*Herpes viruses*	2 (0.46%)	9 (6.82%)	3 (5.36%)
*Adenovirus*	5 (1.16%)	6 (4.55%)	1 (1.79%)
*ParaInfluenza virus*	1 (0.23%)	3 (2.27%)	3 (5.36%)
*Human metapneumovirus*	1 (0.23%)	8 (6.06%)	1 (1.79%)
**Fungus, N (detection rate)**			
*Candida*	0	6 (4.55%)	2 (3.57%)
*Pneumocystis jirovecii*	0	0	6 (10.71%)

### Comparison between pertussis patients with or without *M. pneumoniae* co-infection

**FIGURE 3:**
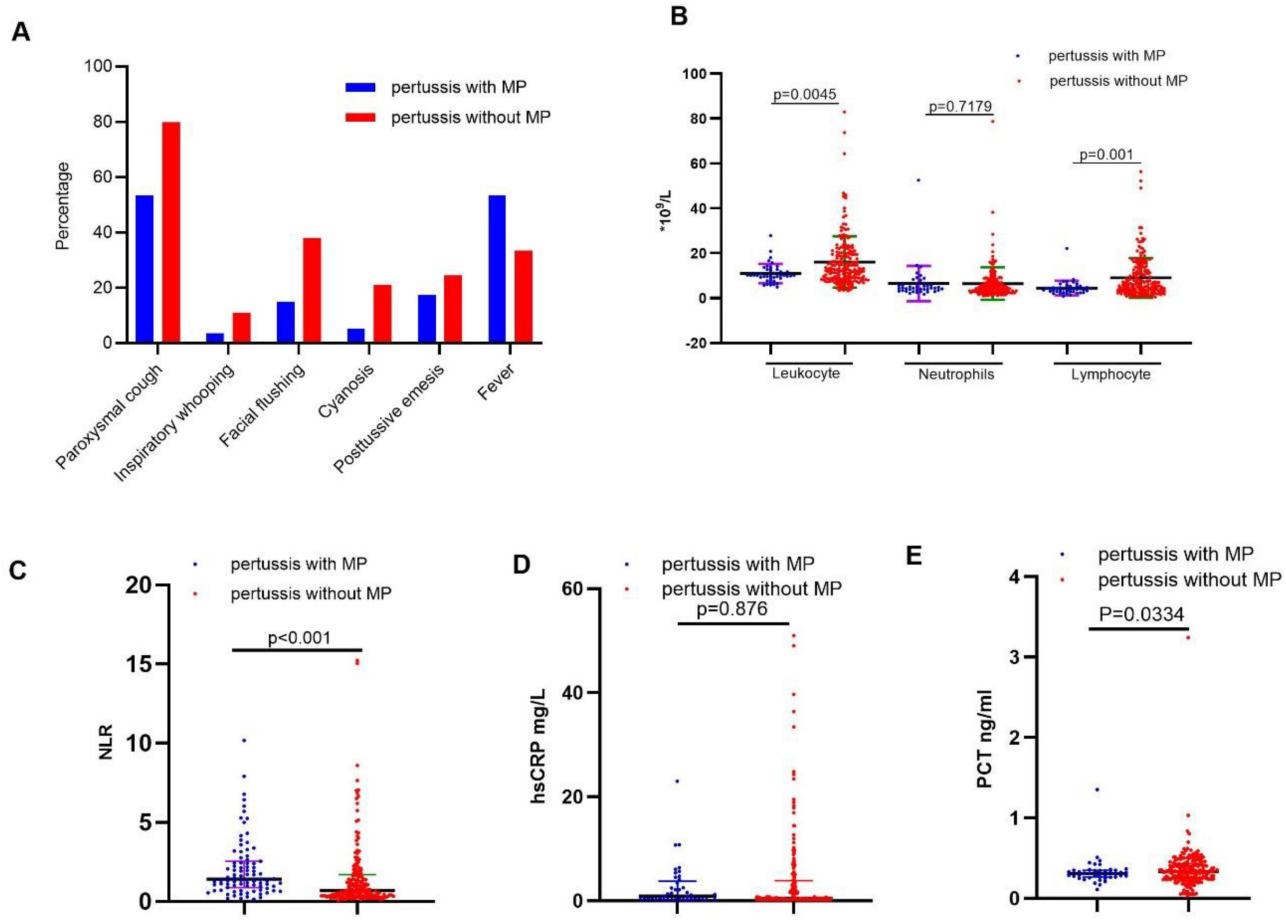
Comparison between pertussis patients with or without *Mycoplasma pneumoniae* co-infection. **(A)** Comparison of clinical characteristics of pertussis patients with or withou*t Mycoplasma pneumoniae* co-infection. **(B)** Comparison of WBC-related parameters of pertussis patients with or withou*t Mycoplasma pneumoniae* co-infection. **(C)** Comparison of NLR of pertussis patients with or withou*t Mycoplasma pneumoniae* co-infection. **(D,E)** Comparison of hsCRP or PCT of pertussis patients with or withou*t Mycoplasma pneumoniae* co-infection.

Compared to children without *M. pneumoniae* co-infection, as depicted in Figure 3A, children with *M. pneumoniae* co-infection experienced a greater incidence of fever and cyanosis but a lower incidence of paroxysmal cough and post-tussive vomiting. Children with *M. pneumoniae* co-infection have notably lower counts of WBC and lymphocytes (Figure 3B), and a significantly higher NLR (Figure 3C) compared to children without *M. pneumoniae* co-infection. Although children with *M. pneumoniae* co-infection exhibited higher hsCRP levels and lower PCT levels than those without *M. pneumoniae* co-infection (Figures 3D,E), these differences were not statistically significant.

## Discussion

This retrospective study analyzed the clinical data of 620 children with whooping cough. Most of the included cases were children over 12 months old (416, 67.09%), followed by infants aged 2–6 months (131, 21.13%). The distribution of age was significantly different from that of other recent studies (Dai et al., 2025; Monchausse et al., 2025; Scutari et al., 2025), where most pertussis patients were aged < 6 months. Variations in the study period and regional factors may have contributed to this discrepancy. Some research suggests that the age distribution of pertussis shifted after the COVID-19 pandemic (Tian et al., 2025), transitioning from being predominantly infants to primarily preschool and school-aged children. Strict epidemic prevention and control measures may reduce infant exposure to *B. pertussis*, resulting in an age shift as susceptible children accumulated. Genotype change *B. pertussis* was considered partially responsible for globally resurge of pertussis. The dominant *B. pertussis* strain shift from *ptxP1* to *ptxP3* (Cai et al., 2023; Mai et al., 2025), which changed its antigen gene alleles to obtain adaptation, may reduce the vaccine’s protective effect. All the above may be parts of the reasons for the increase in infections among preschool and school-aged children. In our analysis, most patients did not exhibit typical whooping cough symptoms (Table 1), which may be related to factors such as age, immune status, history of infection, and disease progression.

In our study, more than 50% of children with pertussis presented with co-infections—a rate consistent with that reported in a Shenzhen-based study (Tian et al., 2025) but lower than rates observed in other investigations (Qing et al., 2025; Scutari et al., 2025). These discrepancies may be attributed to differences in the age distribution of patients with pertussis, detection methods for co-infecting pathogens, and study duration. Our study primarily included children aged > 12 months, a demographic profile similar to that of the Shenzhen study (Tian et al., 2025). In contrast, studies reporting higher co-infection rates predominantly enrolled infants aged < 6 months (Qing et al., 2025). Existing evidence suggests that pertussis patients aged < 6 months are more susceptible to co-infections (Korppi and Hiltunen, 2007; Frassanito et al., 2017). Consistently, we observed a significantly higher rate of co-infections among infants aged < 6 months compared with older children in our cohort. Young infants are more susceptible to coinfections, which may be attributed to the immaturity of their immune system. One study suggests that age-dependent deficiencies in natural killer (NK) cells and interferon-gamma (IFN-γ) function may contribute to severe pertussis infections (Mitchell et al., 2024). Additionally, colonization or infection with Candida species may influence disease progression of COVID-19 by modulating interferon function through IL-17 signaling (Ziegler et al., 2024). Insufficient induction of innate immune responses following pertussis infection in young infants could be a potential factor predisposing them to coinfections.

The impact of co-infections on pertussis remains controversial (Frassanito et al., 2017; Muloiwa et al., 2020; Qing et al., 2025; Scutari et al., 2025). Some studies have indicated that co-infections intensify pertussis symptoms and are associated with adverse outcomes (Muloiwa et al., 2020; Scutari et al., 2025), while others suggest that co-infections may lead to atypical clinical presentations (He et al., 2025), resulting in delayed or misdiagnosis. Conversely, some studies found no significant influence of co-infections on clinical manifestations or disease progression (Frassanito et al., 2017). In our study, pertussis patients with co-infections had significantly higher rates of hospitalization and ICU admission, as well as longer hospital stays, than those with pertussis alone (Table 1). Additionally, co-infected children were more prone to spasmodic coughing and cyanosis and required higher proportions of respiratory support (Table 1). One study has indicated that the cytokine storm in COVID-19 patients can lead to secondary bacterial infections, consequently inducing acute kidney injury (Li X. Q. et al., 2021). Pediatric patients with pertussis co-infections typically present with more pronounced clinical symptoms. However, whether this is associated with a cytokine storm triggered by Bordetella pertussis and/or other co-infecting pathogens warrants further investigation.

Pertussis has traditionally been considered an afebrile or minimally febrile infectious disease (Rubis et al., 2024). We discovered that co-infections resulted in a greater proportion of febrile cases among pertussis patients (Table 1), showing that healthcare providers should not ignore the possibility of pertussis in children with fever during routine clinical practice.

Pertussis is often characterized by elevated peripheral WBC and lymphocyte counts in infants and children (Carbonetti, 2016). In our study, pertussis patients with co-infection exhibited significantly higher WBC and neutrophil than those with pertussis alone, though no significant difference was observed in lymphocyte between the two groups. The NLR was also significantly higher in children with co-infection (Table 1). Neutrophils are key participants in the innate immune system. Studies have shown that neutrophils are involved in clearing Bordetella pertussis during pertussis infection (Borkner et al., 2021). In pertussis with co-infection, the increase in neutrophil counts and NLR is more pronounced compared to those with pertussis only, suggesting that co-infecting pathogens may trigger a more intense immune response.

According to Table 2, children with pertussis with viral co-infection experienced a significantly higher incidence of spasmodic cough, cyanosis, hospitalization, ICU admission, and the need for assisted ventilation compared to those with bacterial co-infections alone. These results suggest that viral co-infections can intensify the clinical symptoms of pertussis, which is consistent with the findings of previous studies (Gan and Wu, 2025; Scutari et al., 2025). Respiratory pathogen co-infection may worsen immune dysfunction and laboratory abnormalities in children with pertussis, potentially contributing to the aggravated severity of the disease. The proportion of viral coinfections in children with pertussis increased after the COVID-19 pandemic (Tian et al., 2025), accompanied by an increase in the incidence of cyanosis, respiratory failure, and other severe manifestations.

Studies have shown that co-infection is independently associated with adverse clinical outcomes of pertussis, including longer hospital stays and more severe complications (Abu Raya et al., 2013; Marshall et al., 2015; Baroudy et al., 2018). The data (Table 3) shows that hospitalization rates and ICU admissions, along with the length of hospital stay, are directly linked to the number of co-infecting pathogens in children with pertussis. Our findings are consistent with those of other researchers who observed that co-infections can intensify the clinical manifestations of pertussis.

In our study, the composition of the co-infection pathogen spectrum was partially consistent with previous domestic and international reports (Muloiwa et al., 2020; Qing et al., 2025). *M. pneumoniae* and *Rhinovirus* were the most common co-infecting pathogens in our study, which is consistent with the findings of previous study (Liu et al., 2020; Poeta et al., 2024). Notably, our study discovered a substantially higher detection rate of *M. pneumoniae* compared to other researchers (Gan and Wu, 2025; Scutari et al., 2025), while the detection rates of *ParaInfluenza virus* and *Adenovirus* were lower than those reported in other studies (Tian et al., 2025). Differences in the respiratory pathogen spectrum in the study region during the study period may account for this variation. The peak of *M. pneumoniae* infections in Guangzhou following the COVID-19 pandemic (Li Y. et al., 2024), may partially explain our results.

Variations in the composition and detection rates of co-infecting pathogens were noted across different age groups (Figure 1; Table 4). Co-infections including *M. pneumoniae*, *Rhinovirus*, *ParaInfluenza virus*, and *Influenza virus A&B* predominantly occurred in pertussis patients over 12 months old, possibly due to the heightened risk of respiratory pathogen transmission among preschool and school-aged children in communal settings such as schools and childcare facilities (Meyer Sauteur et al., 2016; Li Z. J. et al., 2021). In contrast, *Cytomegalovirus* co-infections were mainly observed in younger infants with pertussis, likely due to its transmission routes, primarily vertical transmission and contact transmission (Pontes et al., 2024).

A total of 208 pertussis patients in our study had bacterial co-infections, predominantly caused by Gram-negative bacteria, consistent with previous study (Gan and Wu, 2025). The age distribution varied significantly among different bacterial pathogens. Pathogens associated with community-acquired infections, such as *M. catarrhalis*, *H. influenzae*, and *S. pneumoniae*, primarily caused co-infections in pertussis patients older than 12 months. In contrast, pathogens, such as *A. baumannii* and *K. pneumoniae*, that are prone to causing hospital-acquired infections were more frequently observed in younger infants with pertussis. This age-related distribution pattern may be attributed to disparities in hospitalization experiences and social activities.

Differences were observed in the composition and detection rates of co-infecting pathogens between outpatients and hospitalized inpatients. Patients with pertussis co-infected with *M. pneumoniae* showed clinical symptoms comparable to those with pure pertussis infection and did not need hospitalization, implying that *M. pneumoniae* co-infection does not worsen the clinical symptoms of pertussis. However, a study conducted in South Africa found that co-infections with *M. pneumoniae*, *C. pneumoniae*, and *ParaInfluenza virus* worsened the symptoms of pertussis (Muloiwa et al., 2020). Although the reason for this discrepancy remains unclear, differences in the study populations may contribute to this discrepancy. The South African study specifically focused on patients hospitalized with lower respiratory tract infections (Muloiwa et al., 2020).

Co-infections caused by *H. influenzae* or *S. pneumoniae* was more frequently observed in hospitalized patients or those admitted to the ICU. A study conducted in Italy also found an association between *H. influenzae* co-infection and ICU admission among children with pertussis (Scutari et al., 2025).

The detection rate of *Rhinovirus* co-infection was comparable among children with pertussis across different hospitalization statuses, suggesting that *Rhinovirus* co-infection may not be associated with the severity of pertussis. This finding differs from previous studies, which indicated that *Rhinovirus* co-infection could exacerbate pertussis (Ferronato et al., 2013; Baroudy et al., 2018). The discrepancy in results may be attributed to differences in the age distribution of the study populations, as the average age of participants in our study was higher than that in prior research.

In contrast to rhinovirus, all children with pertussis co-infected with *Cytomegalovirus* and the majority of those co-infected with *Respiratory syncytial virus* required hospitalization or even ICU admission. This finding aligns with previous research (Scutari et al., 2025). Both *Cytomegalovirus* and *Respiratory syncytial virus* primarily cause co-infections in young infants with pertussis, who often have not received or completed full vaccination. Studies have indicated that the inflammatory storm in COVID-19 patients makes them more susceptible to secondary bacterial infections, which in turn can exacerbate their symptoms (Li X. Q. et al., 2021). In infants who have not been vaccinated against pertussis, the lack of vaccine-induced protection allows Bordetella pertussis to potentially trigger a more intense inflammatory storm. This heightened state of inflammation may increase their vulnerability to co-infections with viruses such as cytomegalovirus and respiratory syncytial virus, ultimately leading to more severe clinical manifestations.

Although the number of pertussis cases co-infected with fungi is relatively low, all such cases required hospitalization. Notably, all six pertussis patients co-infected with *P. jirovecii* necessitated ICU admission, indicating that co-infection with *P. jirovecii* may intensify clinical symptoms of the patients.

The spectrum of pathogens causing co-infections in children with pertussis can differ substantially depending on the period and location, with varying effects on clinical manifestations and outcomes due to co-infections with different pathogens. In clinical settings, it is essential to give more consideration to identifying co-infecting pathogens in patients with pertussis, especially those under 6 months old, who may be more susceptible to infections caused by multiple pathogens. Identifying co-infecting pathogens will simplify the creation of more logical and tailored treatment plans, which will decrease the likelihood of negative consequences for children infected with pertussis.

This retrospective study has several limitations. First, we were unable to perform data analysis stratified by vaccination status because vaccination records for the children were unavailable in our information system. This may have introduced a certain degree of bias into our analysis. The clinical presentation of pertussis is significantly influenced by vaccination status, and the immune response to B. pertussis infection likely differs among children with different vaccination histories. These differences in immune response could affect the occurrence of coinfections and the spectrum of co-infecting pathogens. Second, the detection methods for B. pertussis and co-infecting pathogens included culture, PCR, and metagenomic sequencing in our retrospectively collected cases. The sensitivity in detecting pathogens varies across these methods, and the use of multiple detection techniques may introduce some bias. Finally, the majority of cases included in our analysis were outpatients. Due to the difficulty in tracking follow-up data within our information system, we did not analyze patient outcomes in this study.

## Data Availability

The raw data supporting the conclusions of this article will be made available by the authors, without undue reservation.
